# Iron nanoparticle regulate succinate dehydrogenase activity in canola plants under drought stress

**DOI:** 10.1038/s41598-023-36105-4

**Published:** 2023-06-14

**Authors:** Maryam Rezayian, Vahid Niknam, Maryam Arabloo

**Affiliations:** 1 Department of Plant Biology, School of Biology, College of Science, University of Tehran, Tehran, 14155 Iran; 2 Center of Excellence in Medicinal Plant Metabolites, Tarbiat Modares University, Tehran, Iran

**Keywords:** Plant physiology, Plant stress responses

## Abstract

Application of nutrients as nanoparticle (NP) is an operative manner of nutrient supply for plants, especially under stress conditions. The present study was designed to highlight the role of iron NP on drought tolerance and elucidate the underlying mechanisms in drought-stressed canola plants. Drought stress was imposed by polyethylene glycol different concentrations (0, 10 and 15% (W/V)) with or without iron NP (1.5 and 3 mg/l). A comparative study of several physiological and biochemical parameters have been carried out in canola plants treated by drought and iron NP. Stressed-canola plants showed a reduction in growth parameters, whereas iron NP mostly stimulated growth of stressed plants, which was accompanied by reinforcement in defense mechanisms. Regarding impacts on compatible osmolytes, the data revealed that iron NP was able to regulate osmotic potential by increasing protein, proline and soluble sugar contents. The iron NP application was activated the enzymatic defense system (catalase and polyphenol oxidase) and promoted the non-enzymatic antioxidants (phenol, flavonol and flavonoid). Both of these adaptive responses declined free radicals as well as lipid peroxidation and enhanced the membrane stability and drought tolerance of the plants. Enhanced chlorophyll accumulation via induction of protoporphyrin, magnesium protoporphyrin and protochlorophyllide, by iron NP also contributed towards better stress tolerance. Enzymes of Krebs cycle, namely succinate dehydrogenase and aconitase, were induced by iron NP in canola plants grown under drought stress. These results propose a multifaceted involvement of iron NP, through regulation of activity of respiratory enzymes and antioxidant enzymes, production of reactive oxygen species, osmoregulation and secondary metabolites metabolism, in response to drought stress.

## Introduction

Drought, one of the most severe environmental stresses, harmfully influences the development, growth and yield of plants. It poses a threat to food safety and fitness of the population. Drought stress causes an oxidative environment in cells, which interrupts the proteins function and leads cell damage^1^. Plants are obliged to overcome with extreme reactive oxygen species (ROS) production for protection of cell redox homeostasis. Therefore, the increased ROS contents are recognized and restrictively controlled by a series of ROS-suppressing systems. ROS suppressing mechanisms can be categorized into two types: enzymatic and non-enzymatic antioxidant defense mechanisms, which work interactively and synergistically to counteract free radicals^2^. However, plants approve various mechanisms such as osmotic regulation, which could aid sustain cell turgor, water uptake and functions of stomata by inducing large amounts of compatible osmolytes^3^. Plant reactions to drought stress may involve metabolic pathways such as sugar synthesis, photosynthesis and tricarboxylic acid cycle^3^.

Canola (*Brassica napus* L.), a very main oilseed plant, is source of protein-rich meal and vegetable oil and considerably enhances canola generation over the last 35 years, which presently production rate is six times the recorded production in 1980^4^. Canola is sensitive to drought stress in all stages of growth, from germination to seed set. Drought stress influences stomatal conductance, photosynthesis, protein synthesis, transpiration and metabolite accumulation in canola. Drought stress reduced growth parameters such as shoot and root biomass^5,6^.

Nanoparticles have high reactivity than their bulk scale counterparts due principally to the possession of increased surface area and the potential to engineer particular characterizes through coatings and/or functionalization to improve nutrient supply^7^. Iron oxide nanoparticle (Fe_2_O_3_ NP) has high reactivity and large surface area. Iron NP is constant, fewer expensive and less poisonous as compared to several other metallic nanoparticles^8^. Iron NP is efficiently applied in wastewater remediation, medicine, electronics and biosensors^9^. Iron NP can influence shoot elongation, seed germination, root elongation, chlorophyll a content and miRNA expression^10^. Some previous studies showed the role of iron NP in mitigating the adverse effects of drought stress in plants^11–13^. Bidabadi et al.^14^ presented that iron NP induced antioxidant protection by decreasing malondialdehyde (MDA) and hydrogen peroxide (H_2_O_2_) production, and increasing the content of the non-enzymatic antioxidants and the activity of enzymatic antioxidant in *Vitis vinifera* under drought stress.

The main aim of carrying out the present research was to study whether or not iron NP is involved in mitigation of drought stress in canola plants. We hypothesized that application of iron NP could protect the metabolism and cellular functioning of canola by up-regulating the ROS neutralizing enzymes and the osmoregulatory processes. To our knowledge, this is the first report of iron NP impact on canola plants under stress. The study will increase our understanding concerning to the optimization of iron NP custom in field trials, its importance as a maintainable plan for stress improvement and its complex effect on different vital processes through which alleviation of drought stress can be positively reached.

## Results

### Effect of iron NP on the biomass production

**Figure 1:**
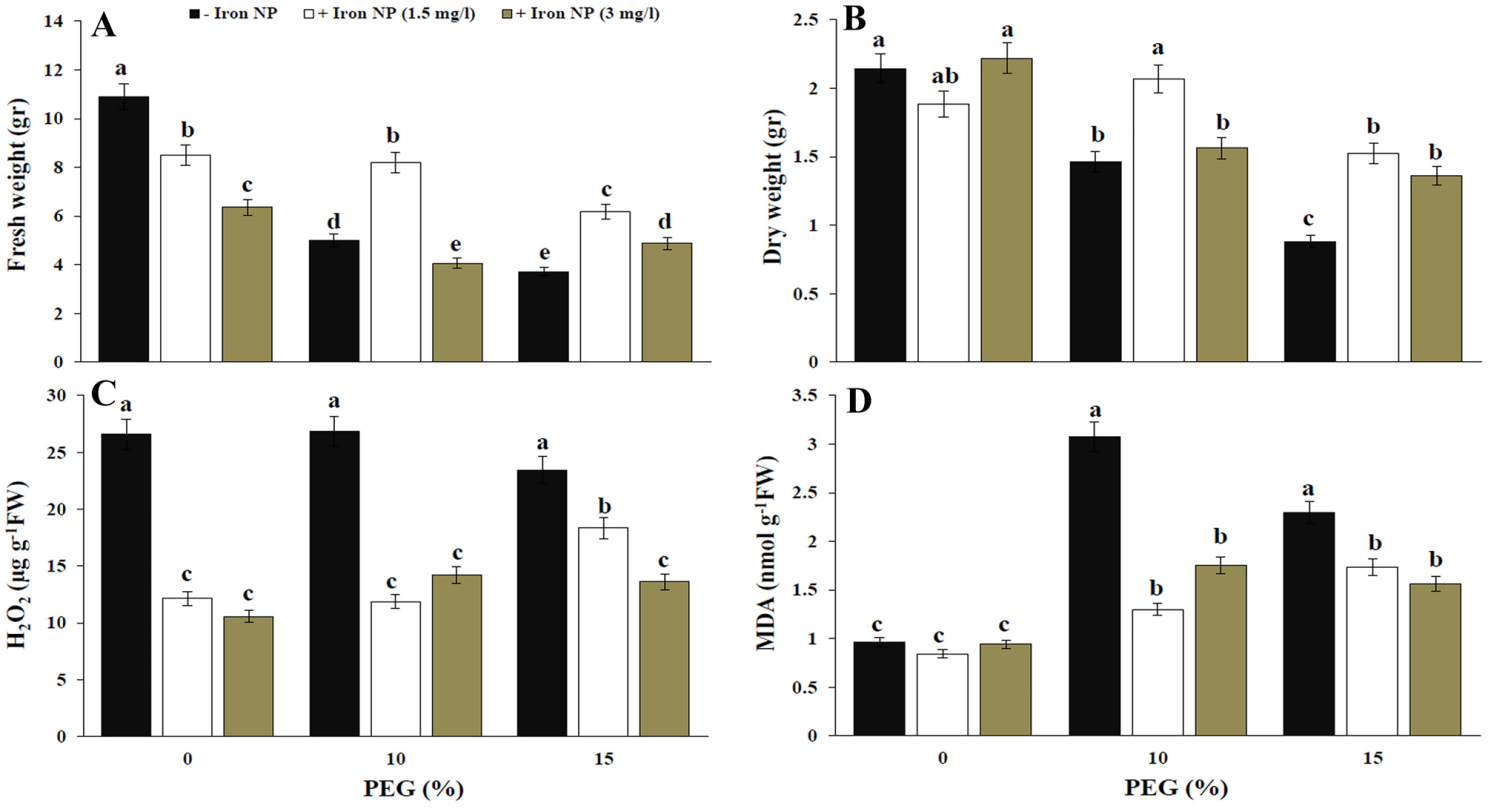
Effect of iron NP under drought stress on fresh weight (**A**), dry weight (**B**), H_2_O_2_ (**C**) and MDA (**D**) in canola plants. Vertical bars indicate Means ± SE based on three replicates. Different letters above columns indicated significant (P < 0.05) differences.

The biomass was determined by measuring the fresh weight and dry weight of plants. These results presented a significant decline in fresh weight and dry weight with increasing drought level. Therefore, the lowest biomass was detected under treatment with high concentration of PEG. These results confirmed the role of drought in the decrease of canola biomass. In contrast, the analysis of the data achieved with the combination treatment of drought with iron NP showed that the iron NP had a significantly positive impact on fresh weight and dry weight of the canola plant. The application of 1.5 mg/l iron NP had the greatest effect on the rise in dry weight; the increases perceived with 1.5 mg/l iron NP were 41.78% and 72.72% in two level of drought, respectively. However, the various concentrations of iron NP differently influenced on the canola growth. These observations detected the positive role of iron NP on growth improvement under drought stress conditions (Fig. 1A,B).

### Oxidative stress markers

No significant change in H_2_O_2_ content was observed following drought exposure. Iron NP exposure reduced the H_2_O_2_ content in plants grown in both control and drought conditions (Fig. 1C). For MDA, the enhance values for 10% and 15% groups were 219.79% and 138.54%, respectively, compared with the corresponding non-drought-exposed plants. Augmented content of MDA is the recognized symbol of oxidative condition caused by drought stress. Under control conditions, no significant change was detected in MDA content as a result of iron NP treatment. The addition of iron NP significantly blocked lipid peroxidation and reduced MDA content in canola plants under drought stress. Also, all tested concentrations of iron NP were found to be effective in decreasing the destructive impact of drought stress. Additionally, this reducing trend was considerable at 10% drought in combination with 1.5 mg/l iron NP (Fig. 1D).

### Enzymatic antioxidant system

The results gained by comparing the average change in antioxidant enzymes (SOD, CAT, POX, and PPO) displayed that there was a significant difference among the different concentrations of drought and iron NP. Activity of SOD enzyme in all the drought treatments was developed by 222.22% and 33.33%, respectively, compared with the plants that were not exposed to drought. Iron NP reduced SOD enzyme activity in an iron NP concentration-dependent manner in stressed plants. Drought stress triggered a significant induction in CAT activity when compared to the control plants; the maximum increase (10.27-fold) in antioxidant activity occurred at 10% PEG. The CAT activity in drought-treated plants following iron NP application was more than that of the control plants, with an increase of 7.18-fold. POX activity showed a significant increase at low level of drought and then a decline with increasing level. Iron NP application elevated the PPO activity in plants grown in both control and drought conditions. The highest PPO activity was perceived in plants co-treated with drought and iron NP. These results showed that iron NP had less effect at the high level of drought (Table 1).

**Table 1:** Impact of iron NP on antioxidant enzymes (U/mg protein) in canola plants under drought stress.

Drought (%)	Iron NP (mg/l)	SOD	CAT	POX	PPO
0	0	0.638 ± 0.011 e	0.115 ± 0.033 d	1.106 ± 0.271 c	0.016 ± 0.001 f
10	0	2.038 ± 0.120 a	1.133 ± 0.221 a	1.757 ± 0.032 a	0.026 ± 0.002 c–e
15	0	0.844 ± 0.110 c	0.165 ± 0.016 cd	1.026 ± 0.113 cd	0.025 ± 0.003 de
0	1.5	0.738 ± 0.202 d	0.161 ± 0.012 cd	1.431 ± 0.211 b	0.027 ± 0.008 cd
10	1.5	0.920 ± 0.073 b	0.794 ± 0.066 b	1.335 ± 0.112 b	0.031 ± 0.005 b
15	1.5	0.563 ± 0.021 f	0.347 ± 0.054 c	0.756 ± 0.121 ef	0.023 ± 0.003 e
0	3	0.641 ± 0.105 e	0.122 ± 0.025 d	0.609 ± 0.052 f	0.025 ± 0.002 de
10	3	0.972 ± 0.101 b	0.115 ± 0.016 d	0.926 ± 0.104 c–e	0.038 ± 0.006 a
15	3	0.690 ± 0.051de	0.109 ± 0.001 d	0.817 ± 0.114 d–f	0.028 ± 0.001 c

Values are means ± SE of three replicates. Different letters indicated significant (P < 0.05) differences.

### Modulation of osmotic regulation by iron NP

**Figure 2:**
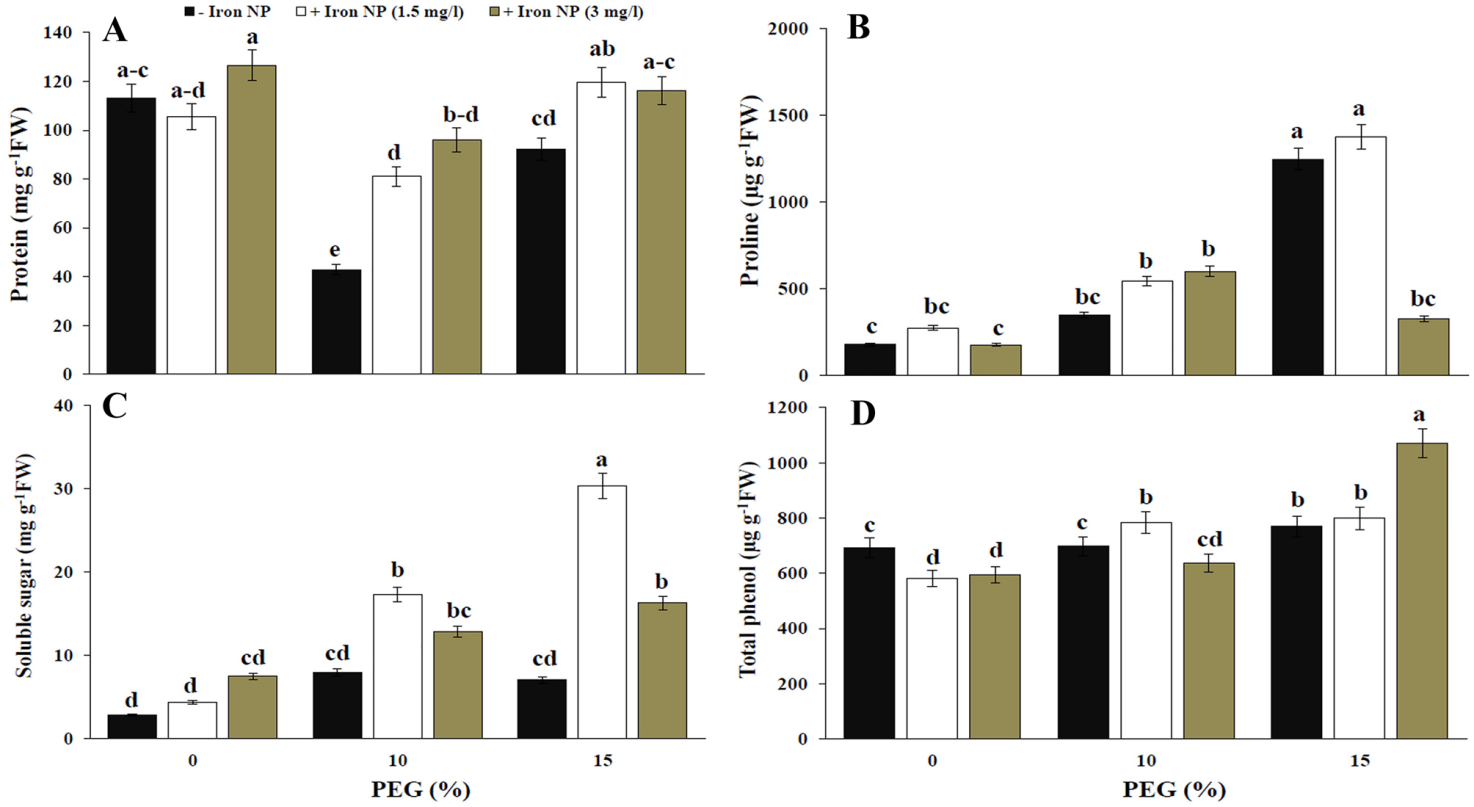
Effect of drought stress and iron NP on protein (**A**), proline (**B**), soluble sugar (**C**) and total phenol (**D**) in canola plants. Vertical bars indicate Means ± SE based on three replicates. Different letters above columns indicated significant (P < 0.05) differences.

Protein content was significantly decreased under drought conditions. Although, iron NP had no impact on protein content in control plants, those plants exposed to low and high drought condition showed a significant rise after iron NP application. Positive effects of iron NP on protein content in low level of drought were more prominent (Fig. 2A). Amount of proline in the plants exposed to 10% and 15% of drought was enhanced by 95.14% and 595.89%, respectively. Proline content in plants co-treated with drought and iron NP was higher than its content plants treated with drought alone. Also, the level of proline in drought-treated plants following iron NP application was more to that of the control plants (Fig. 2B). Significant change in soluble sugar content was not observed in drought-treated plants. Iron NP exposure led to the recovery of soluble sugar content in drought–treated plants. However, application of iron NP resulted in a significant increase in soluble sugar content of low and high drought stresses in an iron NP concentration-dependent manner. The application of 1.5 mg/l iron NP had the greatest impact on the increase in soluble sugar content at high level of drought (Fig. 2C).

### Promotion of non-enzymatic antioxidant defense by iron NP

**Figure 3:**
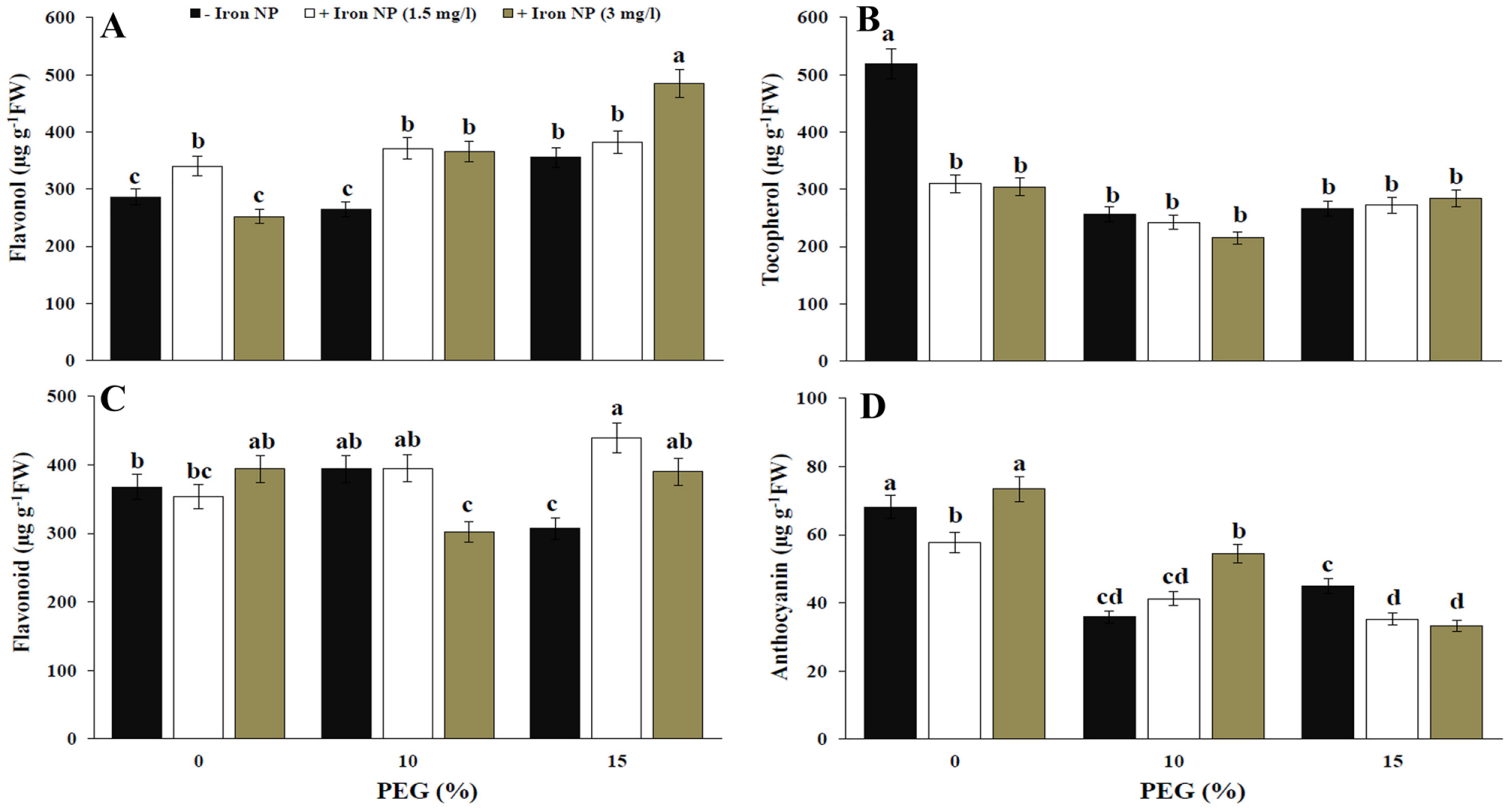
Effect of iron NP application on flavonol (**A**), tocopherol (**B**), flavonoid (**C**) and anthocyanin (**D**) of canola plants grown in drought condition. Vertical bars indicate Means ± SE based on three replicates. Different letters above columns indicated significant (P < 0.05) differences.

High drought level led to a significant induction in total phenol content as compared with control plants. However, this induction was not significant for 10% PEG-treated plants. Considerable increase in total phenol was occurred when plants co-treated with drought and iron NP. Application of 1.5 mg/l iron NP increased total phenol in low level of drought, but 3 mg/l iron NP enhanced this content in high level of drought (Fig. 2D). The content of flavonol in the canola plants that were treated with 15% of drought were heightened by 23.91%, respectively, compared with the untreated plants. The exogenous application of iron NP to drought stressed plants caused higher flavonol content compared with stressed ones. Flavonol content in plants co-treated with drought and iron NP was higher than control plants. Accordingly, the flavonol content was significantly higher in the 15% drought + 3 mg/l iron NP-treated plants than the other treatments (Fig. 3A). Exposure to drought caused a decrease in tocopherol content as compared to unstressed plants. Tocopherol content was reduced by iron NP application in control plants. In plants exposed to drought, tocopherol content were showed non-significant change following iron NP application (Fig. 3B). The flavonoid content relative to the control was significantly inhibited by the application of drought (15%). However, the addition of iron NP to plants exposed to 15% drought significantly improved flavonoid. Exposure to iron NP caused a concentration-dependent increase in flavonoid content. Application of 1.5 mg/l iron NP improved flavonoid content by 43.24% compared with drought treatment (15%). Flavonoid content in plants exposed to 15% drought was more than to the control plants through iron NP treatment (Fig. 3C). Anthocyanin content was considerably declined following plant exposure to drought (Fig. 3D). The anthocyanin content of the stressed plants treated with 3 mg/l was higher than those of drought stressed plants (10%).

### Activity of PAL and respiratory enzymes

**Figure 4:**
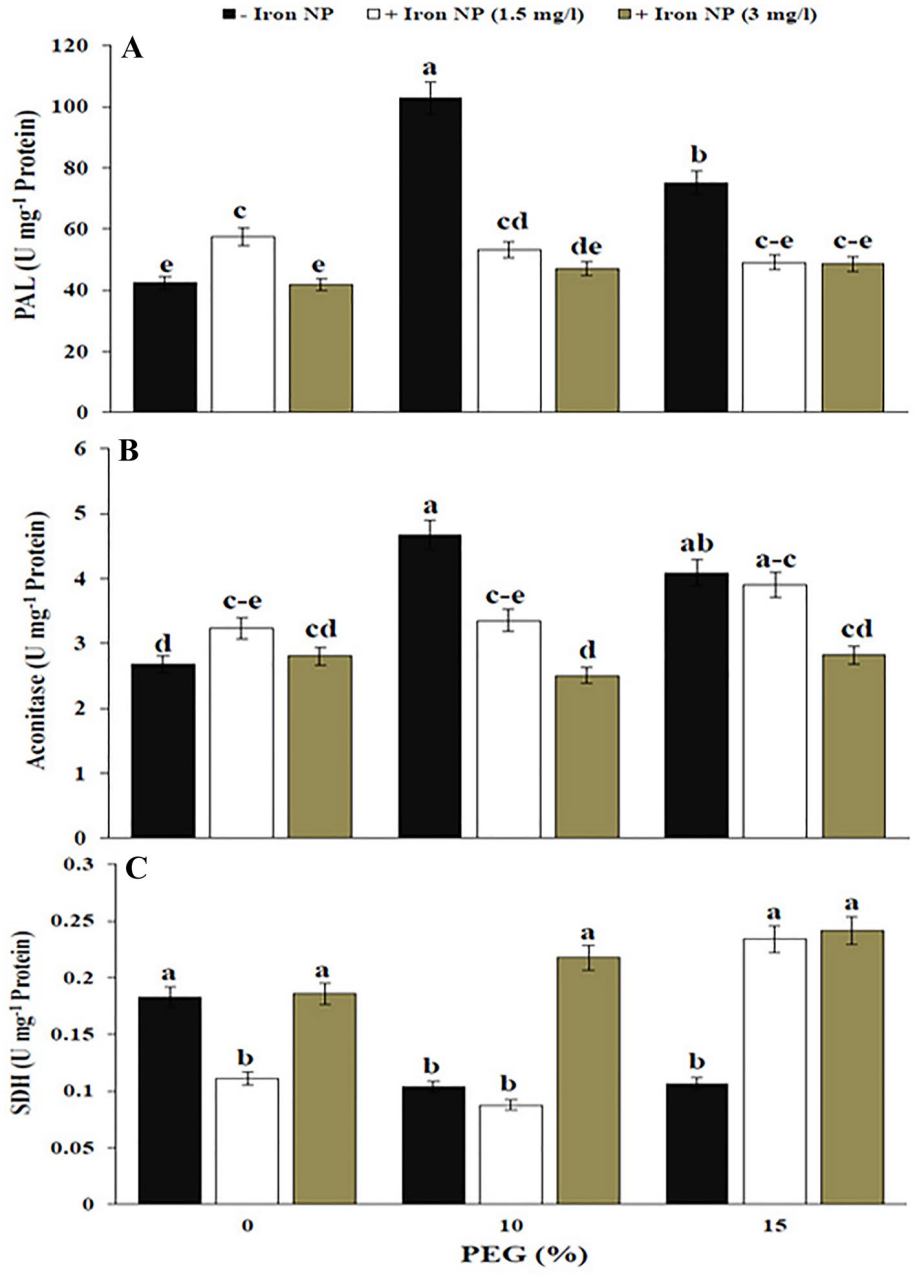
Effect of iron NP under drought stress on PAL (**A**), aconitase (**B**), and SDH (**C**) in canola plants. Vertical bars indicate Means ± SE based on three replicates. Different letters above columns indicated significant (P < 0.05) differences.

The activity of PAL in stressed plants was improved by 142.24% and 77.13%, respectively, with respect to the stress-unexposed plants. Accordingly, the drought-exposed plants in low level of stress showed more PAL activity than high level. The exogenous application of iron NP to the stressed plants decreased the activity of PAL to the non-stressed level (Fig. 4A). Drought stress led to a sharp rise in aconitase activity in canola plants. In plants under drought stress, aconitase activity was 1.74 and 1.52 fold higher compared with those of non-stressed plants, respectively. Application of 1.5 mg/l iron NP increased the aconitase activity in stressed plants compared control treatment. However, the effects of 3 mg/l iron NP were similar to the control treatment for aconitase activity (Fig. 4B). Drought treatment caused a considerable (44.44%) reduction in SDH activity when compared to the control plants; while in drought-exposed plants iron NP supplementation fully restored SDH activity. The highest SDH activity was observed in plants co-treated with drought and iron NP. Iron NP effects were more pronounced in high level of drought than other level. Application of iron NP increased SDH activity by 130% and 27.77% over the drought (15%) and control treatment, respectively (Fig. 4C).

### Photosynthetic pigments and chlorophyll precursors

**Figure 5:**
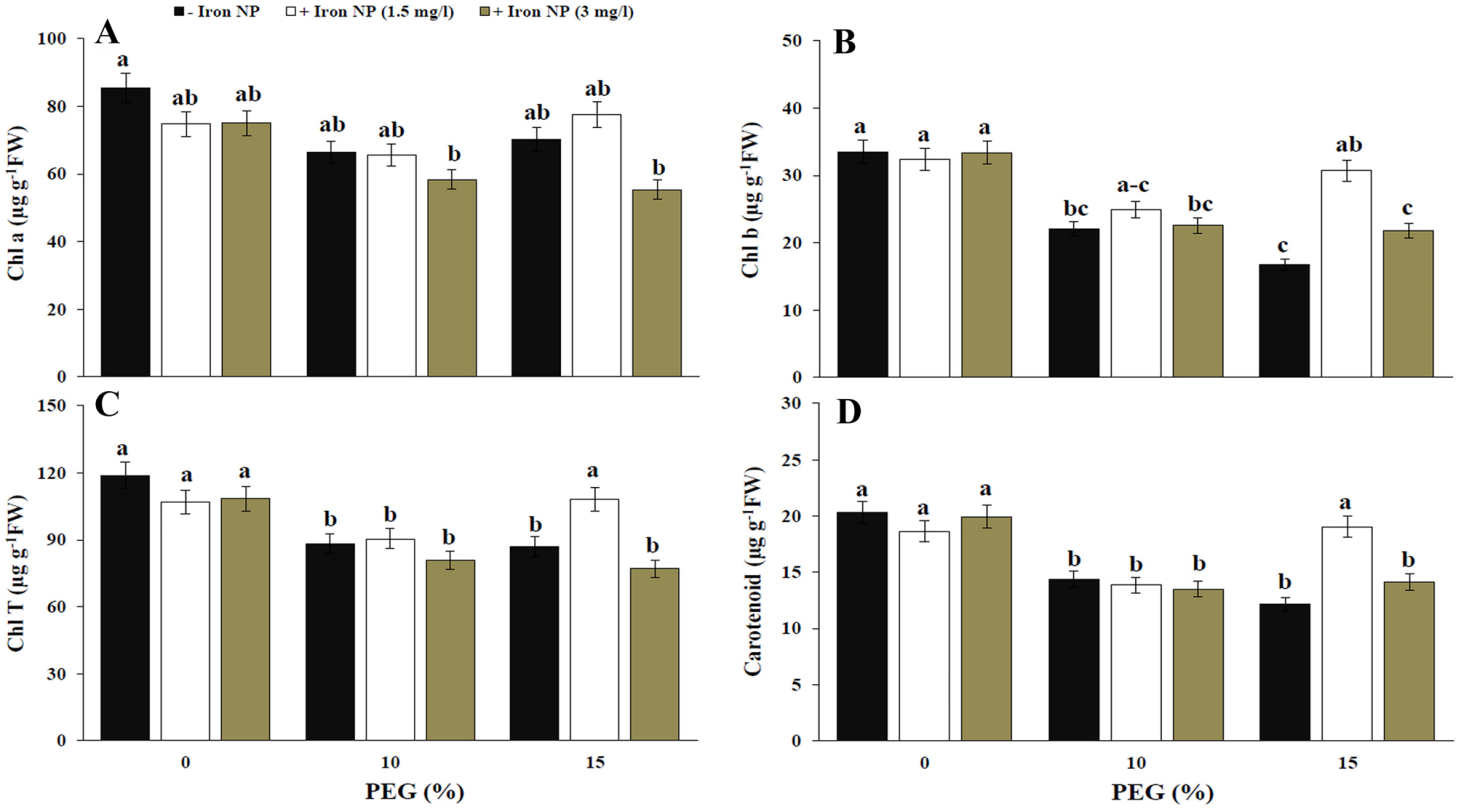
Effect of iron NP on Chl a (**A**), Chl b (**B**), Chl T (**C**) and carotenoid (**D**) of canola plants under different levels of drought stress. Vertical bars indicate Means ± SE based on three replicates. Different letters above columns indicated significant (P < 0.05) differences.

**Figure 6:**
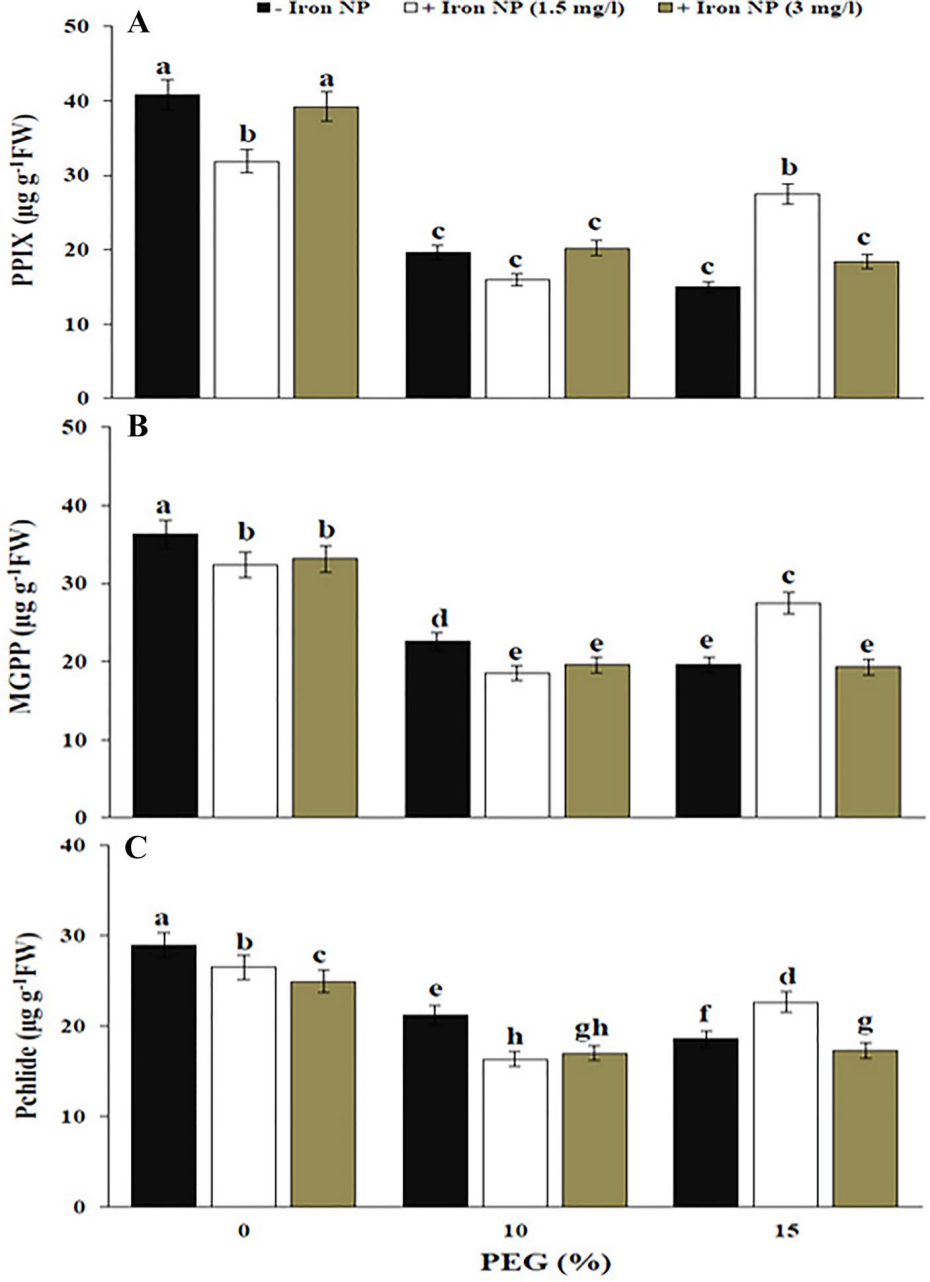
PPIX (**A**), MGPP (**B**) and Pchlide (**C**) of canola plants under drought stress and iron NP. Vertical bars indicate Means ± SE based on three replicates. Different letters above columns indicated significant (P < 0.05) differences.

The results obtained from our photosynthesis index data showed that drought stress significantly decreased the contents of chlorophyll b, total chlorophyll, and carotenoids, which confirmed the negative effects of drought on the metabolism of canola plant. Effect of iron NP on photosynthetic pigments depended on drought level and concentration of iron NP. Application of 1.5 mg/l iron NP in plants exposed to 15% drought enhanced photosynthetic pigments, which induced a 83.84% increase in chlorophyll b content, a 24.43% increase in total chlorophyll content, and a 56.61% increase in carotenoid content (Fig. 5). The drought stress caused 52.07% and 63.50 decrease in the PPIX compared with non-stressed plants, respectively. The exogenous application of 3 mg/l iron NP to drought-stressed plants caused no significant change in the PPIX content of compared with that of stressed plants. Application of 1.5 mg/l iron NP in 15%-treated plants enhanced PPIX content by 84.11% compared drought treatment (Fig. 6A). The results describing the effect of drought on the MGPP and Pchlide content showed a significant linear decline in their contents with increasing drought levels. So, the highest level of damage was detected at high level of drought was significantly higher in the 15%-treated plants than the other level. The reduction of MGPP content was significantly higher than the Pchlide content. Induction in MGPP and Pchlide content was observed in the combination treatment of 15% drought + 1.5 mg/l iron NP (Fig. 6B,C). These data may reflect the undesirable impacts of low iron NP concentration on the chlorophyll precursors.

### Interactions between iron NP, various drought levels and studied parameters through PCA analyze

**Figure 7:**
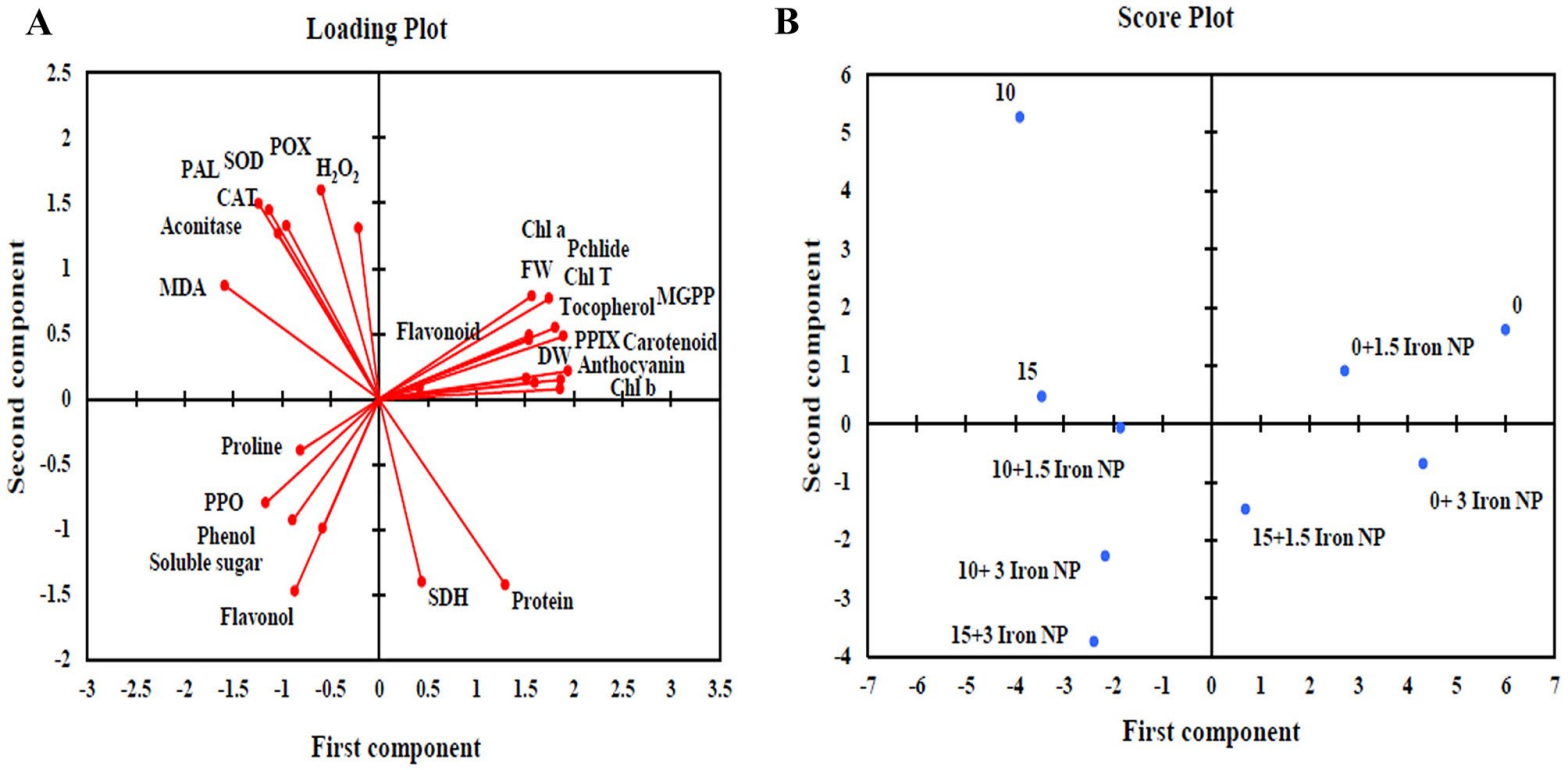
Principal component analysis (PCA) to understand parameter and treatment variability in canola plants. *SDH* Succinate dehydrogenase, *PAL* Phenylalanine ammonia-lyase, *H*_*2*_*O*_*2*_ Hydrogen peroxide, *MDA* Malondialdehyde, *FW* Fresh weight, *DW* Dry weight, *SOD* Superoxide dismutase, *CAT* Catalase, *POX* Peroxidase, *PPO* Polyphenol oxidase, *Chl a* Chlorophyll a, *Chl b* Chlorophyll b, *Chl T* Total chlorophyll, PPIX Protoporphyrin, *MGPP* Magnesium protoporphyrin, *Pchlide* Protochlorophyllide.

PCA (loading and score plot) was performed to study the interaction between different parameters and levels of drought to assess the maximum variability of data and iron NP treatment. The loading plot shown in Fig. 7A of various parameters advocated that growth, chlorophyll precursors, photosynthetic pigments and secondary metabolites (tocopherol, flavonoid and anthocyanin) were positively correlated with each other and negatively with MDA and H_2_O_2_. These findings confirmed that that non-enzymatic defense mechanism is important for cope on destructive effects of stress in canola plants. The score plot (Fig. 7B) represented the approved grouping of different treatments (iron NP and drought levels). The control treatment along with 1.5 mg/l iron NP treatment was considered as the best value giving treatments. Its impact was monitored by that of 15% drought + 1.5 mg/l iron NP. This displays the alleviating impact of iron NP treatment in the presence of drought levels. The drought levels in the absence of iron NP impose severe growth limitations in canola plants as both these treatments were grouped on the upper left-hand side of the score plot. The highest concentration of iron NP with different levels of drought were grouped together in the lower two rectangles of the score plot. There was less alleviation in the presence of the highest concentration of iron NP with drought and this was confirmed by score plot as these treatments were clustered together in a negative component.

## Discussion

Drought stress is a major abiotic stress which typically reduces plant growth and development. To our knowledge, no study thus far has explored the potential of exogenous iron NP application to improve drought tolerance in canola plants. The results of this study show that exogenous iron NP influences the physiological and metabolic responses to drought stress and recognizes some mechanisms that underlie this iron NP-induced tolerance in canola. According to the obtained results, the drought exposure destructively influenced the growth of canola at the vegetative stage; however, this disagreeable impact was more prominent on plants at high level of drought. The diminished biomass is interpreted to be related with the direct and indirect impacts of drought on the mechanisms involved in biomass establishment. Drought stress decreases nutrient uptake and/or transport to the shoot, disturbances the structure of enzymes, reduces photosynthesis, consequently causing nutritional and hormonal discrepancy in the plants. Also, drought stress leads to osmotic stress that can cause to turgor loss, thus, preventing in plant growth and development^15,16^. The present study, however, iron NP improved growth features of the canola plants by mitigating the negative effects of drought. This enhancement might reveal the physiological roles of iron, which is necessary for photosynthesis. The results achieved in our experiment showed that iron NP can enhance biomass as calculated by both fresh and dry weight indexes, reversing the impact of drought on canola plants. It seems that the induces in the antioxidant ability, compatible osmolytes and photosynthetic properties induced by iron NP application play important functions in enhancing plant biomass, with substantial helpful effects at low and high drought levels in combination with iron NP application. The increase in plant growth characteristics because of iron NP application might reflect the constant membrane integrity, as confirmed by the augmentation of antioxidant systems and reduced oxidative injury. Iron was directly participated in the photosynthetic operation of plants and, accordingly, their growth and development. Naturally, approximately 80% of iron is in photosynthetic cells where it is vital for the biosynthesis of cytochromes and other heme-comprising compounds, such as the electron transport system, chlorophyll and the construction of Fe–S clusters^14^. Nano-compounds are rapidly absorbed by plants and provide the plants with essential nutrients. Hence, a rise in plant growth happens with the nanoparticles application^15^. This increase in plant growth is possibly the result of declining abscisic acid due to nano-Fe_2_O_3_ application and following induction of growth and postponing senescence^17^. Linh et al.^18^ displayed that iron NP application can develop drought tolerance of soybean plants by activating drought-associated genes expression. The mechanism underling iron NP-promoted tolerance to drought still is the matter of discussion.

H_2_O_2_ content and lipid peroxidation are main factors that show the level of oxidative injury in plants under critical environmental conditions. The increase in MDA content observed in the drought-treated plants designated that drought stress led to cellular dysfunction by increasing lipid peroxidation. The increase in MDA content was observed in all cases, proposing that drought triggered oxidative hurt to cell membranes in canola leaves. However, the stressed-plants treated iron NP in the present study showed reduced H_2_O_2_ content and lipid peroxidation. These impacts might reveal improved activity of antioxidant enzymes by iron NP treatment. In the current study, a noticeable decline in the MDA content of canola plants, which indicated that drought-induced oxidative damage was alleviated by iron NP. Lipid peroxidation of the cell membrane is one of the most important marks of damage and ionic leakage in stress conditions. Under stress, as a result of lipid peroxidation, cell membranes are injured and the selective permeability of the membrane is disturbed, which causes to higher permeability and electrolyte leakage in the cells^19^. Thus, we suggest that the protective impacts of iron NP are attributable to the decline in plasma membrane permeability and lipid peroxidation of canola cells. The role of iron in electron redox reactions makes it a chief element with an essential function in biological reactions, containing sharp reaction with free radicals, oxygen transportation and electron transportation. Iron induce activity of enzymatic antioxidants in plants, which adds to the alleviation of the impacts of free radicals on membrane organizations^20^. The data achieved from the study of stressed-canola plants showed that iron NP was capable to regulate and scavenge ROS by decreasing H_2_O_2_ levels. The SOD activity is one of the most main ways causing to H_2_O_2_ production, which exchanges dismutase superoxide radicals into H_2_O_2_^21^. In our experiment, iron NP decreased the SOD activity, which caused reduction in H_2_O_2_ content in canola plants under stressful conditions.

Mozafari et al.^22^ reported that the application of iron NP in Grape significantly increased the antioxidant enzymes activities under salt stress. According to our results, the efficiency of the impact of iron NP on antioxidant enzymes activities is related to the concentration of iron NP, drought level and enzyme type. Iron NP application decreased SOD and POX activity, but 1.5 mg/l iron NP enhanced CAT activity al high level of drought. At low level of drought, PPO activity induced by two concentrations of iron NP. In our investigation, iron NP had more effect on CAT enzyme rather than SOD enzyme. This is consistent with our results correlated to H_2_O_2_ content, which exhibited significant reduction after iron NP exposure. Sharma et al.^23^ stated that Fe has a vital function in plant metabolism such as stimulating catalase enzymes. When iron is used in the form of nanoparticles, it can be very suitable in supporting canola plants to readily uptake required amount of iron under drought conditions. The NPs might enhance nitrate reductase activity that is involved in NO synthesis in plants. The NO can then induce antioxidant genes expression, which detoxify the ROS and inhibit lipid peroxidation^23,24^. Askary et al.^25^ demonstrated that iron NP declined SOD activity *Mentha piperita* L. under salinity stress that confirmed our data. Under drought stress, SOD and POX activities were increased in canola plants, but iron NP application declined the activities of these enzymes. This might be due to the detoxification of ROS in a non-enzymatic manner or the augmentation of other antioxidant enzyme activities. These alterations in antioxidant enzymes activities may be responsible for iron NP-related resistance to drought stress in canola. Our results presented an effective correlation between iron NP and oxidative enzymes in plant cells. Although this will open the question that whether iron NP intermediates this connection or its function is expressed by the constituent of this regulatory mechanism?

Under drought stress, proline content increases in plants^25^. Similarly, in our study proline level increased in leaves following plants exposure to drought stress. It has been well recognized that the accumulation of proline could maintain plants against drought stress by suppressing ROS and protecting osmotic balance^26^. The results in Fig. 2A demonstrated that drought stress resulted in a decrement in the protein content in canola plants. Reduction of protein content under drought stress may be caused by the impact of oxidative stress on protein structure. To avoid dangerous dehydration, accumulation of protein, proline and soluble sugar are increased in the plant cells. The obtained data let us to conclude that the studied concentrations of iron NP can positively influence on the content of protein, proline and soluble sugar in canola plants under drought conditions, indicating a role of iron NP in the osmotic regulation in plant cells. According to our findings, effect of iron NP on osmolytes content depended on drought intensity and type of osmolytes. At low level of drought, iron NP leaded more induction proline and protein content, but enhancement of soluble sugar content was prominent at high level of drought. Since iron has role in protein structure and nitrogen metabolism, its application can enhance the protein content^27^. Moreover, the iron NP induces the antioxidant systems and thus inhibits protein degradation. Increased proline content in drought-stressed plants exposed to iron NP may arise in resistance activities. Similarly, proline accumulation was observed in *Zea mays* L. in response to iron NP. Since Fe is involved in enzymatic reactions, thus it may increase proline content^28^. Hyperaccumulation of proline enhances water uptake by inducing osmotic pressure, causing in heightened tolerance to drought stress in plants. Chloroplast is organelle with high demand to iron. Such as, iron-sulfur cluster is the cofactor for some iron-sulfur proteins in photosynthesis^28^. Augmented level of soluble sugar in iron NP treated plants might be the result of photosynthesis induction. However, increased carbohydrate content of wheat were observed by due to the application of nano-Fe oxide^29^.

Phenolic compounds are acting important roles in the defense system of plants against various biotic and abiotic stresses. Phenolic compounds as secondary metabolites can maintain plants under drought conditions. They have antioxidant and free radical suppressing properties. Therefore, they can stop lipid peroxidation and remove reactive oxygen forms when plants are subjected to different stressful conditions^30,31^. According to our findings, impact of drought stress on secondary metabolites was related on metabolite types. Drought stress induced phenol and flavonol content in canola plant; while the content of tocopherol, flavonoid and anthocyanin declined under stress conditions. In conformity with the data of this study, water deficit boosted the total phenolic compounds of *Brassica napus*^32^. PAL has a key role in the biosynthesis pathway of lignins, phenolics and phytoalexins. PAL is an important pointer of stress and can be operative in determining biotic and abiotic stresses^32,33^. The data of our experiment presented that PAL activity augmented with the drought stress. This enhanced activity could be a possible reason for the observed improvement in the phenolic compounds. Application of iron NP improved secondary metabolites in drought-subjected plants. This superiority was more pronounced under severe drought stress. Enhancing secondary metabolites by iron NP treatment can suppress ROS and protect canola plants from harmful effects of drought. So, the increment of secondary metabolites levels can be considered as a drought tolerance improving cause in canola plants by iron NP. Induction in phenolic content under stress is resulted from the stimulation of defense systems in plants in contradiction of oxidative damage from ions. The specification of phenolic compounds is because of their carboxyl and hydroxyl groups which can connect to iron. Phenols can neutralize iron ions by chelating them and decline superoxide amount resulting from Fenton reaction^34,35^. Nourozi et al.^36^ identified that the reason for the more content of flavonoid and phenolic in *Dracocephalum kotschyi* after iron NP treatment is an induction in expression of genes involved in phenylpropanoid pathway. Our findings presented the amount of enzymatic and non-enzymatic antioxidant in canola plants depending on iron NP level. However, by enhancing iron NP level, the activity of antioxidant enzymes is lessened which illustrations inadequacy of enzymatic antioxidant system at high concentration of iron NP. It appears that in canola plants, non-enzymatic system is important for overcome on negative effects of stress. Thus, when the enzymatic defense system is deteriorated, the non-enzyme defense system is stimulated and it aids to eliminate the ROS and their damaging impacts on the plant.

The Krebs cycle is as a central portion of metabolism in all living cells and acts an important defense system under abiotic stress conditions. It protects the carbon pond of the cells and any disorder in the cycle will disturb the carbon cycle, inhibiting the energy production of cells^37,38^. Aconitase catalyzes the citrate to isocitrate in the Krebs cycle. The other important enzyme of Krebs cycle is SDH, catalyzing the formation of fumarate from succinate by releasing one molecule of FADH_2_. In our study, we found a differential response of respiratory enzymes under drought stress. While the Aconitase activity was significantly elevated in canola plants, SDH activity was inhibited under stress conditions. A sharp decline in the SDH activity in canola plants related to metabolic disorders happening during drought stress, resulting in negative impacts on the respiratory metabolism. To date, many investigators have recognized the controlling role of stress in the activity of respiratory enzymes in plants^39,40^. Iron NP treatment escalated the activity of respiratory enzymes in canola plants, thereby augmenting the Krebs chain metabolism. Iron NP has a potential impact on growth elevation in canola plants by enhancing the activity of respiratory enzymes. The higher SDH activity probably converted succinate more efficiently to fumarate which served as the substrate for the next step of Krebs cycle. SDH plays a central function in mitochondrial metabolism by catalyzing the oxidation of succinate to fumarate and also connects the Krebs cycle with electron transfer chain (ETC)^37^. The iron NP-induced increment of SDH in stressed plants might be ascribed to activation of antioxidant system, which caused to considerable reduction of H_2_O_2_ initiating oxidation of thiol groups of enzymes. SDH is the only enzyme that contributes in both Krebs cycle and ETC^38^, so iron NP can influence on Krebs cycle and ETC by influencing SDH enzyme. Aconitase is iron-sulfur protein that participates in Krebs cycle in mitochondria. This enzyme has trans-regulatory role in cytosol that adjusts iron homeostasis at post-transcriptional level^41^. SDH comprises the 3 Fe/S centers that arbitrates electron transfer to ubiquinone^42^. Therefore, because iron is present in the structure of these two enzymes, iron NP application can directly influence on these enzymes.

The drought stress declined the chlorophyll and carotenoid contents in canola plants, also triggered the oxidative stress in these plants which is due to enhance in H_2_O_2_ accumulation. Increasing oxidative stress in canola plants due to drought can reduce photosynthetic pigments that lessened the photosynthetic rate. In addition, according to our results, the reduction in chlorophyll precursors by drought stress could be another reason for chlorophyll diminution. Water stress regulates metabolites of the porphyrin biosynthetic pathway through their scavenging to cope with excited-state dynamics of tetrapyrroles in the plants, subsequently reducing the photodynamic stress imposed by drought. Porphyrin intermediates may act equally a possible shift in ROS and/or dehydration-mediated mechanisms and may be complicated in the complex network adjusting stress-responsive genes. If drought and porphyrin simultaneously initiate the production of ROS to a certain level, the signal could referee the signaling cascade for plants to reach effective down-regulation of porphyrin biosynthetic genes^43^. The present results revealed that the content of chlorophyll, carotenoid and chlorophyll precursors significantly augmented with the application of iron NP. Effect of iron NP on these parameters was concentration dependent because better, chlorophyll, carotenoid and chlorophyll precursors content were witnessed in canola plants treated with low iron NP concentration. The positive effect of minor concentrations of Fe_3_O_4_ NPs on chlorophyll level is correlated to the enhance in photosynthetic carbon assimilation^44^. Our findings showed that iron treatment has increased the chlorophyll content in canola plants by suppressing drought-induced oxidative stress and increasing chlorophyll precursors. The increased chlorophyll content also recommended the promotion of photosynthesis activity in the plants. Iron is essentially found in photosynthetic cells where it is necessary for the synthesis of chlorophyll. Iron oxide NPs can develop the biochemical reactions of chloroplast and thylakoid membranes that cause induction of photosynthetic activity^45,46^. Different enzymatic mechanisms that induce chlorophyll activity need iron, which can be promoted by iron NP application. Thus it is clear that application of iron NP heightens photosynthetic activity, thereby improves plant growth and yield^47,48^. Similarly, it was found that Fe_3_O_4_ NP treatment enhanced chlorophyll content in plants^11,49^. Iron may contribute in the coproporphyrinogenase catalyzed reaction which forms PPIX^50^. Iron requires for the anabolism of MGPP monomethyl ester. Spiller et al.^51^ proposed that iron is necessary in the conversion of MGPP monomethyl ester to Pchlide. This reaction is catalyzed by an iron containing oxygenase. Therefore, considering these concepts, iron NP treatment can influence on the chlorophyll precursors. As our best knowledge, there has not yet been studied the effect of iron NP on chlorophyll precursors. Therefore, these results could initiate more comprehensive studies of plant responses to nanoparticles.

## Conclusion

This study showed that iron NP boosted drought tolerance of canola and provided new strategy for augmenting plant tolerance to drought stress. Our results in this experiment illuminated for the first time the efficacy of exogenous iron NP in combating drought-induced disruption in Krebs cycle of canola plants by promoting respiratory cycle for induction of drought stress tolerance. This study also revealed the potential role of iron NP in regulating osmotic status and antioxidant capacity that caused to restoration of cellular homeostasis during drought stress. However, our results explain a wide range of functions, which powerfully suggests a central role for iron NP, in plant planned responses to drought stress. There is inadequate information concerning the positive impacts of iron NP on physiological mechanisms that arise in plants under drought stress, but the data of the present investigate provide a new platform for discovering the intervention of iron NP in physiological, biochemical and molecular responses in plants.

## Materials and methods

### Plant cultivation and chemical treatments

All the plant experiments were in compliance with relevant institutional, national, and international guidelines and legislation. Seeds of canola (*B. napus*) CV. RGS003 were obtained from the Seed and Plant Improvement Institute of Karaj, Iran. Seed collection was done with permission. Seeds were sown in each plastic pot containing perlite. Plants were grown at average day/night temperatures of 25/18 °C. The pots were irrigated with equal amount of half strength Hoagland solution for three week. Drought stress and iron NP were applied during vegetative growth of plants. Drought stress was imposed by polyethylene glycol (PEG) different concentrations (0, 10 (− 0.15 MPa, moderate level of drought) and 15 (− 0.3 MPa, severe level of drought) % (W/V)) with or without iron NP (1.5 and 3 mg/l) for three weeks at alternative days. 50 ml of half strength Hoagland solution containing PEG was given to each pot. The concentrations of selected iron NP were observed on the pervious works of Hoang et al.^52^, Mozafari et al.^22^ and Plaksenkova et al.^11^. Each treatment was carried out in triplicate. The experiment was laid out in a randomized complete block design. Three weeks after treatment, all leaves from plants were collected and immediately frozen in liquid nitrogen before being stored at − 70 °C for analyses in all the experiments.

After 21 days of planting, fresh weight was measured. Dry weight was obtained by drying samples in an oven for 24 h at 60 °C until constant weight.

### Determination of antioxidant enzymes

Leaf material (0.5 g) was homogenized at 4 °C with 1 M Tris–HCl (pH 6.8) to estimate different enzyme activities. The homogenate was centrifuged at 13,000 rpm for 20 min at 4 °C and the obtained supernatant was kept at − 70 °C and later used for enzyme assays. Protein content was measured according to Bradford^53^ using bovine serum albumin as the standard.

Catalase (CAT) activity was assayed based on Aebi^54^. The reaction mixture comprised 50 mM phosphate buffer (pH 7.0), H_2_O_2_ (3%) and 10 µl enzyme extract. The decline in absorption was followed for 180 s and CAT activity was expressed as unit per mg of protein.

The activity of superoxide dismutase (SOD) was assayed based on Giannopolitis and Ries^55^ procedure. Reaction solution included potassium phosphate buffer (50 mM), 0.1 mM EDTA, 13 mM methionine, 75 μM NBT, 75 μM riboflavin and 100 μl of enzyme extract. The reaction solution was placed in front of the light for 18 min and then absorbance was measured at 560 nm.

For measurement of peroxidase (POX) activity, reaction mixture consisted 0.1 ml benzidine (40 mM), 0.2 ml H_2_O_2_ (3%), 2 ml of 0.2 M acetate buffer (pH 4.8) and 50 μl of enzyme extract. Activity of this enzyme was recorded at 530 nm^56^.

Polyphenol oxidase (PPO) activity was determined according to Raymond et al.^57^. The reaction mixture contained 2.5 ml of 200 mM potassium phosphate buffer (pH 6.8), 0.2 ml of 20 mM pyrogallol and 20 μl enzyme extract. The enzyme activity was recorded at 430 nm.

### Hydrogen peroxide and lipid peroxidation

H_2_O_2_ content was estimated by Velikova et al.^50^ method. Leaf tissue (0.5 g) was homogenized in 0.1% trichloroacetic acid (TCA) then was centrifuged at 12,000 rpm for 15 min. Supernatant (0.5 ml) was added to 0.5 ml potassium phosphate buffer (pH 7.0) and 1 ml potassium iodide (1 M) and absorbance was recorded at 390 nm.

MDA content was determined through Heath and Packer^51^ procedure. Leaf tissue (0.5 g) was homogenized in 0.1% TCA and then was centrifuged at 13,000 rpm for 10 min. The supernatant (0.5 ml) was mixed with 1 ml of thiobarbituric acid (0.5%) in 20% TCA. The mixture was heated in 95 °C for half-hour and then was centrifuged at 13,000 rpm for 15 min. The absorbance of supernatant was recorded at 532 and 600 nm.

### Measurement of proline and soluble sugar

Leaf tissue (0.05 g) was homogenized in 5 ml sulfosalicylic acid (3%) and then was centrifuged at 13,000 rpm for 20 min. Proline content was measured according to Bates et al.^52^. 2 ml of supernatant was mixed with acid ninhydrin (2 ml) and glacial acetic acid (2 ml) and then was boiled at 100 °C for 1 h. The reaction mixture was extracted with 4 ml toluene and the absorbance was recorded at 520 nm.

The soluble sugar content was assessed by Dubois et al.^58^ method. Fresh leaf tissue (0.1 g) was homogenized in 3 ml distilled water and then was centrifuged at 5000 rpm for 20 min. The supernatant (500 µl) was mixed with 450 µl distilled water, 500 µl phenol 5% and 2.5 ml sulfuric acid 97% and then the absorbance was assayed at 485 nm after 30 min.

### Assay of phenolic compounds

In order to preparation of methanolic extract, 0.1 g of dry tissue was homogenized in 5 ml methanol 80% and then was centrifuged at 5000 rpm for 20 min. For the total phenol content measurement, 0.1 ml methanolic extract was mixed with 2.5 ml Folin-Ciocalteu reagent 10%. The mixtures were neutralized by sodium bicarbonate 7% and then absorbance was recorded at 765 nm^54^.

The Akkol et al.^54^ method was used for flavonol content measurement. In this method, 0.5 ml aluminum chloride 2% and 1.5 ml sodium acetate 5% were added to 0.5 ml methanolic extract and absorbance of was recorded at 445 nm after 2.5 h and rutin was used as a standard.

Tocopherol content was estimated according to the method of Kayden et al.^55^. Approximately 0.1 g of leaf was homogenized in 3 ml of ethanol. One ml of the ethanol extract was pipetted into a 4 ml glass test tube for analysis. The extract was mixed with 0.2 ml of 0.2% bathophenanthroline in ethanol and the content of each tube were thoroughly homogenized. The assay proceeded rapidly from this point and care was taken not to expose the solutions to direct light. 0.2 ml of 1 mM FeC1_3_ solution in ethanol was added, followed by mixing with a Vortex mixer. After 1 min, 0.2 ml of 1 mM H_3_PO_4_ solution in ethanol was added and the contents of the tubes were again thoroughly mixed. The absorbance of the solutions was determined at 534 nm using α-tocopherol as standard and expressed in µg g^-1^ fresh weight.

Content of flavonoid was measured by aluminum chloride method^59^. In this method 1 g of plant material was homogenized in 2 ml of methanol 80%. Methanolic extract (0.5 ml) was mixed with 1.5 ml of methanol, 0.1 ml of aluminium chloride (10%), 0.1 ml of potassium acetate (1 M) and 2.8 ml of distilled water and the absorbance was measured at 415 nm after 30 min.

Anthocyanin content was determined in 0.3% HCl in methanol at 25 °C using the extinction coefficient (33 cm^2^/mol) at 550 nm^60^.

### Phenylalanine ammonia-lyase activity

Assay of Phenylalanine ammonia-lyase (PAL) was performed using the method of Berner et al.^61^ at the optimal pH 8. The reaction mixture consisted of 0.5 ml of enzyme extract and 150 mM of l-phenylalanine was adjusted to 3 ml with the borate buffer. Incubation was done at 40 °C for 30 min. PAL activity was determined at 290 nm, following the formation of *E*-cinnamic acid. Specific activity of enzymes was expressed as micromoles *E*-cinnamic acid formed per min per mg of protein.

### Respiratory enzymes activity

The aconitase activity was measured based on Murthy et al.^62^. The reaction solution contained 20 μl of enzyme extract, phosphate buffer (pH 7, 220 μmole) and citrate (30 μmole). The activity of this enzyme was determined by the increase in the extinction values at 240 nm with citrate.

To succinate dehydrogenase (SDH) activity assay, the reaction solution contained 0.6 ml of phosphate buffer 0.2 M (pH 7.5), 0.2 ml succinate 0.2 M, 0.3 ml KCN 0.01 M and 20 μl enzyme extract. The enzyme activity was recorded at 410 nm^62^.

### Pigment content and chlorophyll precursors

Total chlorophyll, carotenoids and chlorophyll precursors content in leaves were estimated using the method of Yang et al.^63^. The fresh plant material (0.1 g) was extracted using 80% acetone by mortar and pestle. The absorbance of the solution was read at different wavelengths using spectrophotometer. The photosynthetic pigments and precursors were expressed as mg g^−1^ FW.

$$\begin{array}{*{20}l} {{\text{Chl a }}\left( {\upmu {\text{g}}/{\text{ml}}} \right) = { 12}.{\text{25 A}}_{{{663}.{6} }} - { 2}.{\text{55 A}}_{{{646}.{6}}} ,} \hfill \\ {{\text{Chl b }}\left( {\upmu {\text{g}}/{\text{ml}}} \right) = { 2}0.{\text{31 A}}_{{{646}.{6} }} - {4}.{\text{91 A}}_{{{663}.{6}}} ,} \hfill \\ {{\text{Chl Total }}\left( {\upmu {\text{g}}/{\text{ml}}} \right) = { 17}.{\text{76 A}}_{{{646}.{6}}} + { 7}.{\text{34 A}}_{{{663}.{6}}} ,} \hfill \\ {{\text{Car }}\left( {\upmu {\text{g}}/{\text{ml}}} \right) = { 4}.{\text{69 A}}_{{{44}0.{5}}} - \, 0.{\text{267 Chl total,}}} \hfill \\ {{\text{PPIX }}\left( {\upmu {\text{g}}/{\text{ml}}} \right) = { 196}.{\text{25 A}}_{{{575}}} - { 46}.{\text{6A}}_{{{59}0}} - { 58}.{\text{68 A}}_{{{628}}} ,} \hfill \\ {{\text{MGPP }}\left( {\upmu {\text{g}}/{\text{ml}}} \right) = { 61}.{\text{81 A}}_{{{59}0}} - { 23}.{\text{77 A}}_{{{575}}} - { 3}.{\text{55 A}}_{{{628}}} ,} \hfill \\ {{\text{Pchlide }}\left( {\upmu {\text{g}}/{\text{ml}}} \right) = { 42}.{\text{59 A}}_{{{628}}} - { 34}.{\text{22 A}}_{{{575}}} - { 7}.{\text{25 A}}_{{{59}0}} .} \hfill \\ \end{array}$$

In the above formulas, Chl a, Chl b, Chl Total, Car, PPIX, MGPP and Pchlide are chlorophyll a, chlorophyll b, total chlorophyll, carotenoid, protoporphyrin, magnesium protoporphyrin and protochlorophyllide, respectively.

### Statistical analysis

Each experiment was repeated three times and the data were analyzed by using one-way ANOVA using SPSS (version 21). Means were compared by Duncan’s test at the 0.05 level of confidence. Principal component analysis (PCA) analysis was used for obtaining correlation matrix, giving the Pearson’s correlation coefficients between each pair of variables, i.e., the analytical parameters tested. PCA analysis was done by XLSTAT (2016).

## Data Availability

All data generated or analyzed during this study are included in this published article.
